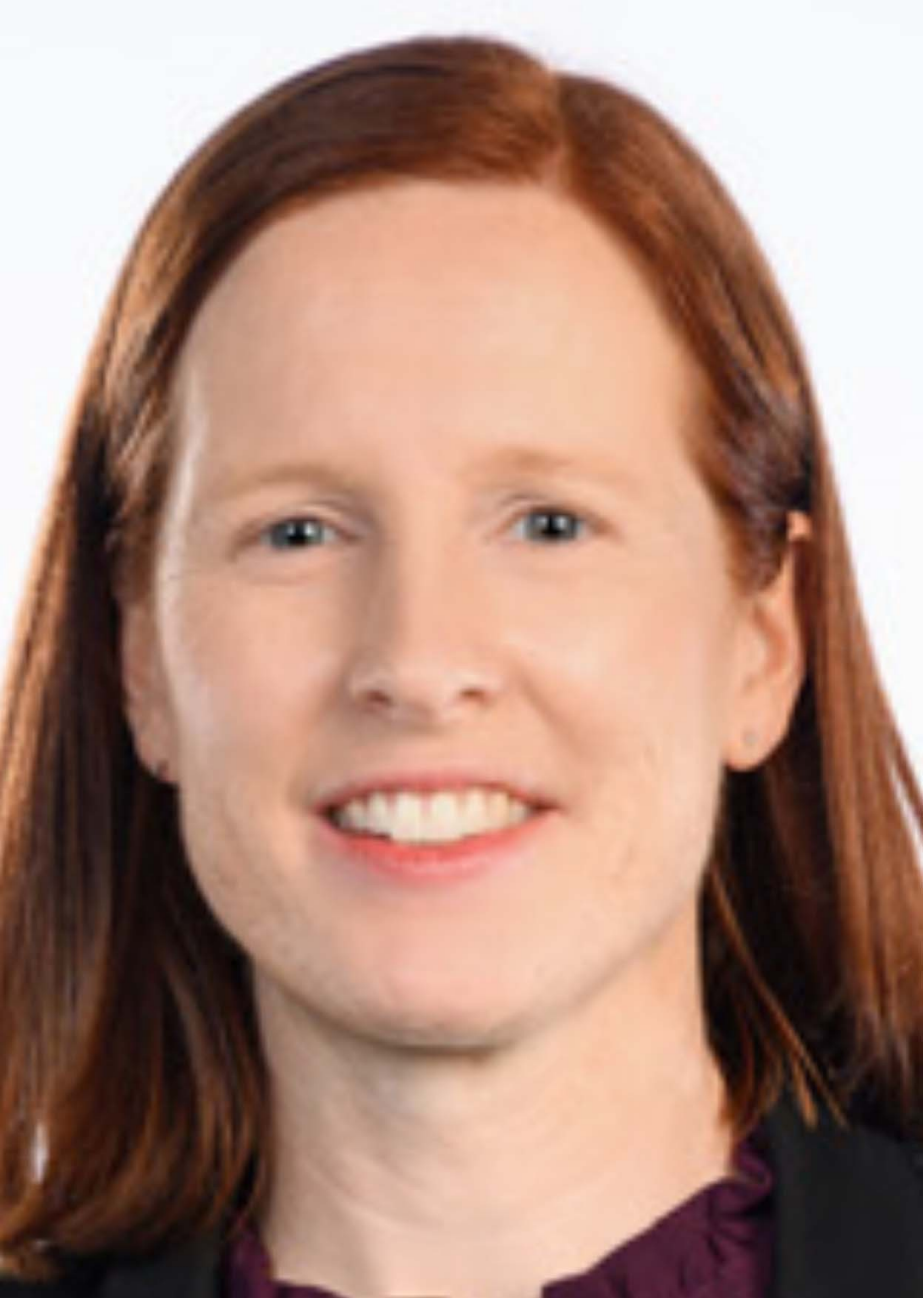# Strategies to Improve the Potency of Oxazolidinones towards Bacterial Biofilms

**DOI:** 10.1002/asia.202200201

**Published:** 2022-04-13

**Authors:** Audrey R. N. Ndukwe, Sandra Wiedbrauk, Nathan R. B. Boase, Kathryn E. Fairfull‐Smith

**Affiliations:** ^1^ School of Chemistry and Physics, Faculty of Science Queensland University of Technology Brisbane Queensland 4001 Australia; ^2^ Centre for Materials Science Queensland University of Technology Brisbane Queensland 4001 Australia

**Keywords:** Antibiotics, Biofilm, Drug delivery, Drug Design, Oxazolidinone

## Abstract

Biofilms are part of the natural lifecycle of bacteria and are known to cause chronic infections that are difficult to treat. Most antibiotics are developed and tested against bacteria in the planktonic state and are ineffective against bacterial biofilms. The oxazolidinones, including the last resort drug linezolid, are one of the main classes of synthetic antibiotics progressed to clinical use in the last 50 years. They have a unique mechanism of action and only develop low levels of resistance in the clinical setting. With the aim of providing insight into strategies to design more potent antibiotic compounds with activity against bacterial biofilms, we review the biofilm activity of clinically approved oxazolidinones and report on structural modifications to oxazolidinones and their delivery systems which lead to enhanced anti‐biofilm activity.

## Graphical abstract

**Biofilms play a key role in chronic infections** yet they are highly resistant to antibiotic treatment. One approach to develop new anti‐biofilm agents is to modify the structure of existing antibiotics to target biofilms. In this review, we provide insight into strategies used to design more potent oxazolidinone compounds with activity against bacterial biofilms and systems used for their delivery.

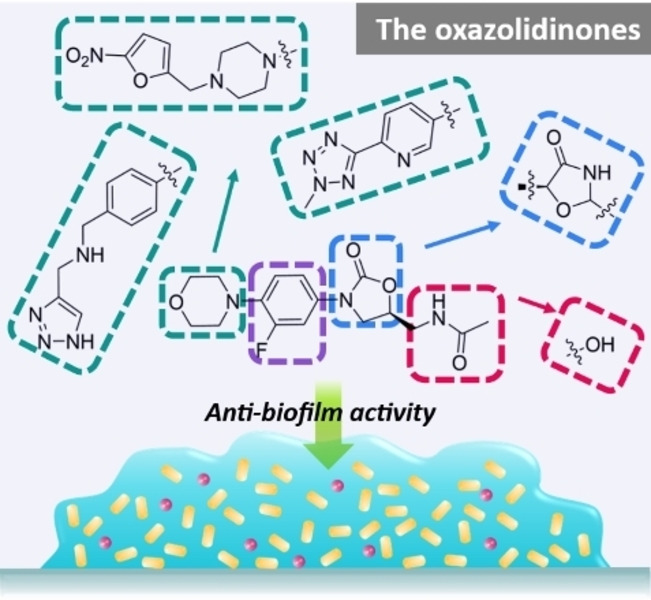

## 1 Introduction

### 1.1 Infectious diseases and biofilms

Infectious diseases are a major cause of mortality worldwide and it is estimated that 17 million people die each year from bacterial infections.^[1]^ Biofilm associated infections are particularly problematic because they are a significant contributor to bacterial pathogenicity, resistant to antibiotic treatment and have significant financial implications, with annual healthcare costs in the United States alone in the billions of dollars.[^2^, ^3^] In humans, biofilms are responsible for up to 80% of all microbial infections^[4]^ and are implicated in diseases such as endocarditis^[5]^ and urinary tract infections.^[6]^ They also occur with scar tissue, wounds, and medical devices such as catheters or joint prostheses, resulting in chronic infections that can be debilitating, and in some cases life threatening.^[7]^

Biofilms are structured associations of bacteria covered by an extracellular polymeric substance (EPS) consisting of extracellular DNA, protein, and polysaccharides.^[8]^ Whilst a common depiction of bacteria is that of a free‐floating planktonic cell, 80% of bacteria exist in the biofilm mode of growth, including clinically important pathogens such as *Staphylococcus aureus, Pseudomonas aeruginosa*, *Escherichia coli* and *Enterococcus faecium*.[^9^, ^10^, ^11^, ^12^, ^13^] Evolutionary pressure has resulted in bacteria forming biofilms as they provide a protected mode of growth. Biofilms enable bacterial cells to weather hostile environments, such as nutrient scarcity, altered pH and mechanical force. They also protect bacteria from the host's immune cells and antibiotic therapy.[^14^, ^15^] Biofilms are a key component of the lifecycle of bacteria, and it is these protective features that can make infections difficult to treat, with biofilms playing a key role in drug resistant infections.^[15]^

**Figure 1:**
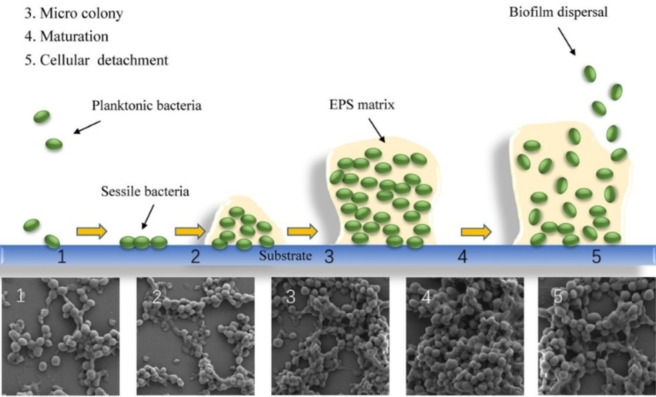
The five stages of biofilm formation depicted pictorally and with microscope magnification. Reproduced with permission. Ref. [24] Copyright 2020, Springer Nature.

Biofilm formation (Figure 1) is a dynamic process that begins when planktonic bacteria irreversibly adhere to a surface. The attached cells start to secrete an EPS and form microcolonies until the maximum cell density is reached. The cells coordinate through quorum sensing (QS) systems, which is the process by which bacteria communicate with each other to form structures such as biofilms.^[16]^ They can use QS to regulate the bacterial population's behaviour such as acquiring nutrients and sensing the density of the bacterial population.[^17^, ^18^] Signals are transmitted by the use of molecules called autoinducers.^[19]^ The type of QS system used in biofilms can be categorised in accordance with the type of signalling molecule used in the system. The signalling molecule acyl homoserine lactone (AHL) is primarily used by gram‐negative bacteria, autoinducer‐2 (AI‐2) is used by both gram‐negative and gram‐positive bacteria while autoinducing peptides (AIP) are used by gram‐positive bacteria.^[20]^ After the biofilm reaches maturity, microcolonies can be released from the biofilm (dispersal) in response to environmental stressors such as nutrient scarcity, oxygen tension, pH or temperature.^[21]^ These microcolonies revert to the planktonic mode of growth and spread the infection to other areas where biofilm formation can recommence.[^22^, ^23^] Biofilms are clinically important and their presence in infection settings exacerbates antibiotic tolerance.

### 1.2 Antibiotic treatment of biofilm infections

Most antibiotics are developed and tested against bacteria in the planktonic state. While planktonic bacteria may be susceptible to antibiotics, it has been shown that bacteria in biofilms can be up to 1000 times more tolerant to antibiotics.[^8^, ^25^] There are many factors thought to influence biofilm antibiotic tolerance (BAT), however these factors do not appear to affect the tolerance for all antibiotics, equally. The composition of the EPS acts as a barrier to cell penetration and slows down the diffusion of drugs.^[26]^ While some antibiotics struggle to penetrate biofilms, others have been found to effectively penetrate; for example tetracycline and ciprofloxacin were found to diffuse throughout uropathogenic *Escherichia coli* and *Klebsiella pneumoniae* biofilms respectively.^[27]^

Even if antibiotics can reach and kill bacteria within a biofilm, they fail to completely eradicate biofilms as persister cells can remain.^[28]^ Persister cells within the biofilm are dormant, metabolically inactive, and incredibly resistant to antibiotics.[^29^, ^30^] When bacterial cells are subjected to environmental stress such as antibiotic treatment, they can enter a dormant state.[^31^, ^32^] This reduced growth rate is thought to influence BAT as many antibiotics target metabolic processes and bacterial cells that reproduced rapidly.[^33^, ^34^] The microenvironment in the deeper layers of a biofilm is characterised by low levels of oxygen and nutrients.^[35]^ It is well known that bacterial growth is slowed down by an inadequate supply of oxygen and nutrients.[^36^, ^37^] Again, dormant or slow growing cells within the hypoxic regions of a biofilm would be less susceptible to antibiotics.[^8^, ^28^] Once the period of stress is over, persister cells can resume growing and repopulate the biofilm. Therefore, the presence of persister cells can lead to relapsing biofilm infections.[^28^, ^31^, ^38^]

### 1.3 The oxazolidinone class of antibiotics

A range of approaches to target biofilms are in development such as hybrid drugs,[^39^, ^40^, ^41^, ^42^, ^43^] antimicrobial peptides,[^44^, ^45^, ^46^, ^47^] and quaternary ammonium compounds.[^28^, ^48^, ^49^, ^50^] Current clinical treatment of biofilm infections still largely relies on antibiotics.[^51^, ^52^, ^53^] The highly antibiotic resistant characteristic of biofilms requires potent antimicrobial agents and novel anti‐biofilm strategies. While combination therapies utilizing antibiotics are a viable strategy to treat biofilm related infections,[^54^, ^55^, ^56^, ^57^, ^58^] one approach to develop new anti‐biofilm agents is the structural modification of existing antibiotics.

The oxazolidinone class of antibiotics is an attractive target to modify in order to enhance biofilm efficacy.[^59^, ^60^] Linezolid is a last resort antibiotic and currently has low levels of resistance in the clinical setting.^[61]^ It is reserved as a last line of defence against bacterial gram‐positive pathogens that cause life threatening and drug resistant infections and are only used when other treatments fail.^[62]^ The World Health Organisation is urgently calling for new antimicrobial development to tackle ‘critical priority’ pathogens such as methicillin resistant *Staphylococcus aureus*.^[63]^ Oxazolidinones are promising candidates as they uniquely target the bacterial ribosome preventing protein biosynthesis and have been shown to have no cross resistance with other antibiotics.^[64]^ If new oxazolidinones can be developed with better potency against biofilms, it will prolong their clinical use. With this goal in mind, this review will focus on clinically approved oxazolidinones, emerging oxazolidinones derivatives and oxazolidinone delivery systems, that have been tested against bacterial biofilms.

## 2 An overview of biofilm testing methods

Before the efficacy of oxazolidinones against biofilms can be discussed, the different methods used to evaluate anti‐biofilm activity must be understood. Several methods can be used to assess the effectiveness of a drug on a biofilm, and it is important to understand that each technique is limited in the information it provides about the biofilm. Here, we introduce some of the methods used to evaluate the efficacy of oxazolidinones against biofilms that are relevant to this review.

The microtiter plate assay can be used to study the early stages of biofilm formation as well as determining the minimum inhibitory concentration (MIC) and minimum bactericidal concentration (MBC) via broth microdilution. Sterile broth culture is used to fill a 96‐well microtiter plate. The next step involves inoculation of the bacteria followed by incubation typically for 24–48 hours. Any planktonic bacteria are removed by rinsing the wells and any biofilms that have been formed are left behind in the wells and stained. The optical density of the biomass is then measured.[^65^, ^66^, ^67^] Dyes can be used to differentiate between dead and live cells.^[68]^ Microtiter assays are well suited to high‐throughput screening as they are relatively simple to execute and enable multiple tests to be done simultaneously.^[69]^ A disadvantage of using the microtiter assay as a model to study biofilms is that the drug is often added during the inoculum stage.^[70]^ This is before the biofilm has adhered to the plate. This means that is it difficult to differentiate between the inhibition of planktonic bacteria, the inhibition of early‐stage biofilms or eradication of mature biofilms.^[71]^

Given the limitation with the traditional microtiter plate, an alternative way to determine the effect of a drug on a biofilm is with a Calgary biofilm device. The device can be used to evaluate the 3D structure of a biofilm over time[^72^, ^73^] as well as ascertain the antibiotic susceptibility of a biofilm through the minimum eradication concentration (MBEC) value.^[74]^ A 96‐well plate is used with a lid containing polystyrene pegs and, unlike the microtiter plate assay, biofilms are grown on the pegs instead of in the wells. The lid containing the formed biofilms is then transferred into a plate containing the drug to be evaluated. After incubation, the biofilms can be displaced from the pegs by sonication for analysis with dyes or viable cell counts (colony forming units – CFUs), or whole pegs can be removed, and the biofilm structure visualised using confocal laser scanning microscopy or scanning electron microscopy. For image analysis, dead or live biofilms are expressed as a percentage or in terms of their optical density.[^74^, ^75^] Like the microtiter assay an MBEC assay is suitable for high‐throughput screening.[^76^, ^77^, ^78^] However, there is some conjecture in the literature as to the definition of MBEC, some define it as the complete eradication of the entire biofilm,[^78^, ^79^] while others define it as eradication of bacteria in the biofilm mode of growth in comparison to growth controls.^[80]^

The Calgary biofilm device and microtiter plate are examples of closed evaluation systems.^[78]^ Biofilms are grown under batch conditions and there is no flow of nutrients in or out of the system.^[81]^ However, they may not accurately replicate *in vivo* conditions where there is a constant flow of fluids such as the flow of blood in catheter related infections.^[73]^ The flow cell is an example of an open evaluation system. Open systems try to replicate the dynamic conditions of *in vivo* biofilm growth and give greater control over growth parameters.[^78^, ^82^] The flow cell assay can be used to study the latter stages of biofilm development and determine whether a drug causes dispersal or eradication of mature biofilms. In this assay, a flow cell chamber is sterilised and inoculated with bacteria. The chamber is left to settle without flow for a short period of time, then a medium is slowly pumped through the system. The biofilm is allowed to grow for 48–72 hours, then the antimicrobial to be tested is added to the medium and slowly pumped through the system. The resulting biofilm can be analysed using dyes, CFU counts, or confocal laser scanning microscopy.[^68^, ^83^] An advantage of flow cell use is that thicker biofilms can be grown than those grown in a Calgary biofilm device and biofilms can be monitored in real time without destruction. The structural parameters of the biofilm such as the thickness, roughness and biomass can be measured using confocal microscopy and analysed with software packages, like Comstat.^[78]^ Unfortunately, flow cell assays are technically challenging to assemble and very time consuming to run.[^73^, ^78^]

Once a biofilm has grown or been treated with a potential antimicrobial, it needs to be quantified. A common technique widely employed for quantification in a microtiter plate or flow cell assay is to measure a biofilm according to its biomass. Crystal violet (CV) is a dye extensively used for biofilm quantification which works by binding negatively charged molecules.[^84^, ^85^] As a result crystal violet stains both dead and live cells along with components that are present in the biofilm matrix such as extra‐cellular DNA, polysaccharides, protein and lipids.[^86^, ^87^, ^88^] While CV is suitable for high‐throughput analysis, due to the inability to differentiate between dead and live cells, a crystal violet assay may not provide accurate information on biofilm eradication but will assess total biofilm mass.

Other dyes such as the fluorogenic dyes SYTO 9 and propidium iodide (PI) can be used to selectively stain components of the biofilm biomass. Whilst both dyes specifically bind to DNA, STYO 9 can passively diffuse through cell membranes, whereas PI is only able to penetrate cell membranes that have been damaged.[^89^, ^90^] When these dyes are used in combination, PI reduces SYTO 9 which causes cells with damaged membranes to be stained red instead of green.[^91^, ^92^] While this technique provides some information on the ratio of dead to live cells, it is important to note that DNA present in the biofilm matrix will also be stained red, which may result in an overestimation of the number of dead biofilm cells measured.^[93]^ Fluorogenic dyes are also limited to the mode of action of the antimicrobial. Dead biofilm cells may still be reported as live if the cell membrane is intact at the time of analysis.^[92]^ It has been suggested that SYTO 9/PI assays should be combined with cell viability assays to confirm the results.^[94]^

As an alternative to quantifying biofilms by their biomass, the metabolic activity of a biofilm can be used instead. The tetrazolium salt dye XTT (2,3‐bis‐(2‐methoxy‐4‐nitro‐5‐sulfophenyl)‐2*H*‐tetrazolium‐5‐carboxanilide) is commonly used as well as the dye resazurin. Both dyes are modified by metabolically active biofilm cells into coloured products (formazan and resorufin respectively).^[89]^ The products can be tracked over time and therefore XTT assays are useful in time kill analysis (where the efficacy of the antimicrobial is determined over time and different points of bacterial growth) as they are less laborious than traditional count plating.^[95]^ However, XTT assays have been reported to have less sensitivity and may be inaccurate in biofilms.[^96^, ^97^, ^98^] This is due to the heterogeneity of biofilm microenvironments. The multiple layers of the biofilm result in biofilm cells with different levels of metabolic activity. Cells in the deeper layers of the biofilm are often dormant which can potentially lead to inaccurate results.^[99]^

Another way to differentiate between dead and live biofilm cells is through a viable cell count also known as a plate count or colony forming units (CFUs) and this approach is typically used to quantify MBEC assays. CFU assays are conducted without the use of dyes. Biofilms cells are collected (usually from a 96‐well plate) and suspended in a liquid medium. Using serial dilution, cells are separated on an agar plate to grow colonies, thus differentiating between dead and live cells. The number of colonies are then counted and compared to the initial number of cells. A plate count does not take into account all the viable cells as some cells, although viable, will be non‐culturable and therefore not recovered and counted.^[69]^ The CFU assay requires a significant amount of biofilm to observe the difference in colony number with some of the biofilms produced in 96 well plates present in too small quantities to use this technique.^[100]^ When plate counts are used to determine the effect of an antimicrobial treatment sometimes carryover occurs. Carryover arises when the antibiotic is present in sufficient concentrations during agar plating to inhibit the biofilm cell growth. This overestimates the activity of the compound tested as the concentration at which eradication occurs will be much lower.^[101]^ Plate counts are also both labour and time intensive.^[69]^ All of the techniques discussed in this section have their advantages and disadvantages and it is important for the results of anti‐biofilm assays to be interpreted carefully.

## 3 Biofilm activity of oxazolidinones approved for clinical use

### 3.1 Linezolid

**Figure 2:**
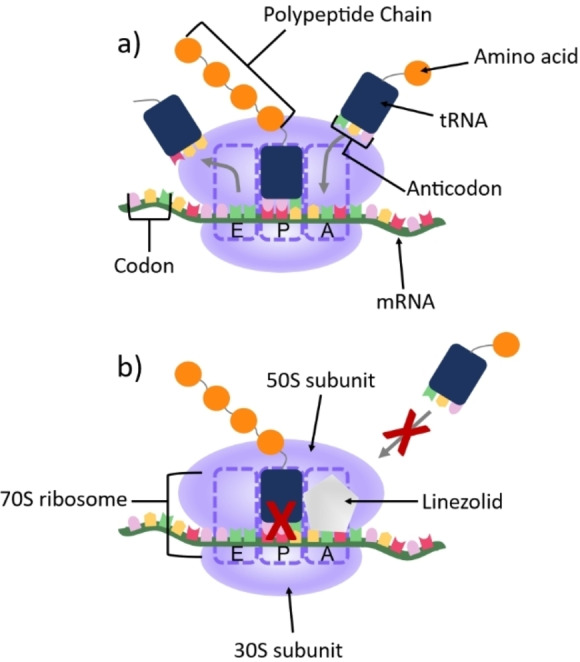
Linezolid's mode of action: a) During the translation of mRNA the ribosome moves along the mRNA, reading codon by codon, to synthesise a chain of amino acids which then fold into protein; b) Linezolid binds to the A site on the 50S ribosome preventing tRNA from binding and therefore inhibiting protein synthesis.

First discovered in the 1990s by the Pharmacia and Upjohn Company,^[102]^ linezolid (trade name Zyvox) was approved for treating pneumonia, skin infections and vancomycin resistant *Enterococcus faecium* infections in 2000 by the US Food and Drug Administration (FDA).^[103]^ Linezolid exhibits a bacteriostatic effect on most gram‐positive bacteria^[104]^ and interacts with the 50S subunit of the prokaryotic ribosome as a part of its mechanism of action (Figure 2). Linezolid binds to the A‐site of the peptidyl transferase centre (PTC) within the 50S subunit.^[105]^ This prevents the positioning of initiator‐tRNA which inhibits formation of a functional 70S ribosome from the 50S and 30S subunits.[^106^, ^107^, ^108^] If the 70S ribosome has already been formed, the bound linezolid prevents tRNA from binding to the A‐site preventing the translation of mRNA into protein.^[109]^ The outcome of both scenarios is the inhibition of protein synthesis, which prevents the bacteria from growing or replicating. While the mechanism of action against planktonic bacteria is well understood, the mechanism of linezolid against biofilms is currently unknown.

**Figure 3:**
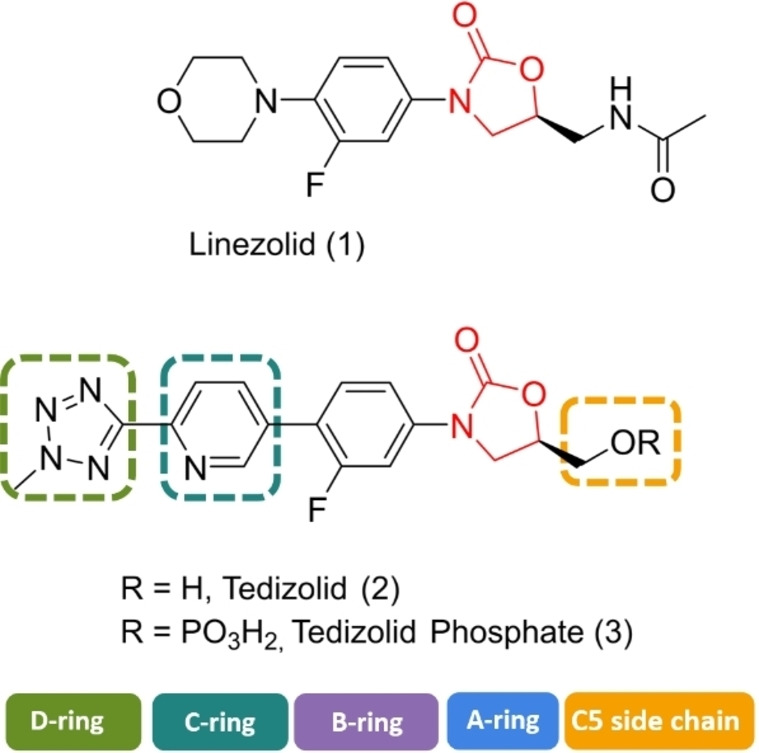
Chemical structures of linezolid (1), tedizolid (2) and tedizolid phosphate (3).

A number of studies have explored the anti‐biofilm activity of linezolid (Figure 3) (Table 1). Tsang and co‐workers^[80]^ investigated the anti‐biofilm activity of linezolid and a range of antibiotics commonly used for periprosthetic joint infections against *Staphylococcal* biofilms. The replacement of joints with prosthetics are extremely commonplace.[^110^, ^111^] These prosthetic joints can become infected and the treatment of these infections is tricky due to their resistance to antibiotics as discussed in section 1.2.[^110^, ^112^] The MBEC values were established for linezolid, rifampicin, clindamycin, vancomycin, ciprofloxacin, daptomycin and gentamicin and were determined against methicillin susceptible *Staphylococcus aureus* (MSSA)‐*N* and coagulase‐negative *Staphylococcus* (CNS‐*J*). Gentamicin and daptomycin were found to be the only effective antibiotics achieving eradication concentrations of 256 and 16 mg/L in MSSA‐*N* respectively and 32 mg/L in CNS‐*J*. The MBEC for all other antibiotics including linezolid was reported as 2048 mg/L for all strains, therefore making them clinically ineffective. When linezolid was used in combination with gentamicin against CNS‐*J* the eradication concentration dropped to 512 mg/L. However, no difference was seen in MSSA‐*N* as the concentration remained at 2048 mg/L. This may suggest that linezolid has an antagonistic effect on gentamicin. The other bacteriostatic antibiotics rifampicin and clindamycin also had an antagonistic effect. It is also important to note that this study focused short exposure times to the antibiotics (3 hours) and longer exposure times may have different outcomes.

**Table 1:** MIC and biofilm assay data of oxazolidinones tested against bacterial biofilms.

Compound	MIC (strain)	Biofilm Assay			Fold Potency vs Linezolid^[a]^	Ref.
		Crystal Violet	MBEC	XTT		
Linezolid (1) 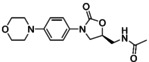	1 μg/mL (MRSA ATCC 43300)	29.2% inhibition at <1 μg/mL		60.8% reduction in metabolic activity at 10× MIC	–	[114]
	2 μg/mL (MSSA ATCC 29213)	29.4% inhibition at <2 μg/mL		32% reduction in metabolic activity at 10× MIC	–	
	2 μg/mL (MRSA 1 clinical isolate)	51.6% inhibition at <2 μg/mL		76% reduction in metabolic activity at 10× MIC	–	
	2 μg/mL (MRSA 2 clinical isolate)	77.3% inhibition at <2 μg/mL		61% reduction in metabolic activity at 10× MIC	–	
	2 μg/mL (Range of clinical *S. aureus* and *S. epidermidis*)		891 μg/mL		–	[131]
	1.5 μg/mL *(S. aureus* 6850 and 2 clinical *S. aureus* isolates)		>2000 μg/mL		–	[120]
	0.5–2 μg/mL (range of MSSE and MRSE strains)	≈85% inhibition at 16x MIC (MRSE ATCC 35984)			–	[132]
	8–16 μg/mL (range of linezolid‐resistant *E. faecalis* clinical isolates)	inhibition at 8x MIC			–	[123]
	2 μg/mL (*S. epidermidis* ATCC 35984)	99.7% inhibition of biofilm adherence at 2 μg/mL			–	[133]
	1 μg/mL (*S. epidermidis* ATCC 35983)	99.7% inhibition of biofilm adherence at 2 μg/mL			–	
	2 μg/mL (MSSA ATCC 25923)		512 μg/mL		–	[134]
	2 μg/mL (MRSA ATCC 33591)		256 μg/mL		–	[134]
	2 μg/mL (MRSA ATCC 43300)		256 μg/mL		–	
	2 μg/mL (Range of clinical MRSA isolates)		256 μg/mL		–	
	0.42±0.06 μg/mL (MIC50) (MRSA USA300‐0114)	approx. 20% of biofilm remaining at 256 μg/mL			–	[135]
	0.95±0.10 μg/mL (MIC90) (MRSA USA300‐0114)	approx. 20% of biofilm remaining at 256 μg/mL			–	
	2–16 μg/mL (MSSA ATCC 29213, range of clinical MSSA isolates including linezolid sensitive and resistant strains)			45% mean cell survival at 10× MIC^[b]^	–	[82]
	2–8 μg/mL (range of clinical MRSA isolates including linezolid sensitive and resistant strains)			45% mean cell survival at 10× MIC^[b]^	–	
	1–512 μg/mL (range of clinical *S. epidermidis* isolates including linezolid sensitive and resistant strains)			≈80% mean cell survival at 10× MIC	–	
	2 μg/mL (MRSE 879)	76% inhibition at 1× MIC^[c]^			–	[136]
	2 μg/mL (MRSA 1026/99)	63% inhibition at 1× MIC^[c]^			–	
	2 μg/mL (*S. epidermidis* ATCC 35983)	86.7% inhibition at 1× MIC^[c]^			–	
Tedizolid (2) 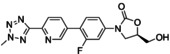	0.25 μg/mL (*S. aureus* 6850 and 2 clinical *S. aureus* isolates)		>675 μg/mL		MIC: 6 MBEC: 3	[120]
	1–4 μg/mL (range of linezolid‐resistant *E. faecalis* clinical isolates)	biofilm inhibition at 8× MIC			MIC: 4–8 CV: 4–8	[123]
	0.125–2 μg/mL (MSSA ATCC 29213, range of clinical MSSA isolates including linezolid sensitive and resistant strains)			≈30% mean cell survival at 10× MIC^[b]^	MIC: 16–8 XTT: 1.3	[82]
	0.125–0.5 μg/mL (range of clinical MRSA isolates including linezolid sensitive and resistant strains)			≈30% mean cell survival at 10× MIC^[b]^	MIC: 16 XTT: 1.3	
	0.125–0.5 μg/mL (range of clinical *S. epidermidis* isolates including linezolid sensitive and resistant strains)			35% mean cell survival at 10× MIC	MIC: 8–1024 XTT: 3	
RBx 11760 (4) 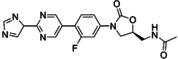	0.25–4 μg/mL (range of MSSE and MRSE strains)	≈95% inhibition at 16× MIC (MRSE ATCC 35984)			MIC: 2–4 CV: 2–4	[132]
FYL‐67 (5) 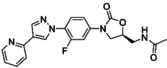	1 μg/mL (MSSA ATCC 25923)		256 μg/mL		MIC: 2 MBEC: 2	[134]
	0.5 μg/mL (MRSA ATCC 33591)		128 μg/mL		MIC: 2 MBEC: 2	
	0.5 μg/mL (MRSA ATCC 43300)		128 μg/mL		MIC: 2 MBEC: 2	
	1 μg/mL (Range of clinical MRSA isolates)		128 μg/mL		MIC: 2 MBEC: 2	
Ranbezolid (6) 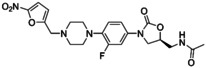	0.25 μg/mL (*S. epidermidis* ATCC 35984)	100.1% inhibition of biofilm adherence at 0.125 μg/mL			MIC: 8 CV: 16	[133]
	0.25 μg/mL (*S. epidermidis* ATCC 35983)	100% inhibition of biofilm adherence at 0.125 μg/mL			MIC: 4 CV: 16	
	0.5 μg/mL (MRSE 879)	93% inhibition at 1× MIC^[c]^			MIC: 4 Safranin: 4–5	[136]
	2 μg/mL (MRSA 1026/99)	91% inhibition at 1× MIC^[c]^			MIC: 1 Safranin: 1.44	
	0.25 μg/mL (*S. epidermidis* ATCC 35983)	85% inhibition at 1× MIC^[c]^			MIC: 8 Safranin: 8	
Radezolid (7) 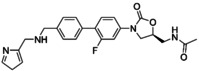	0.5–1 μg/mL (range of linezolid‐resistant *E. faecalis* clinical isolates)	biofilm inhibition at 8× MIC biofilm eradication at 8× MIC			MIC: 16 CV: 1 (inhibition)	[123]
YXL‐13 (11) 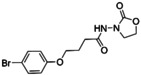	>1112 μg/mL (*P.aeruginosa* POA1)	40.39% inhibition at 162.5 μg/mL			–	[17]
	>1112 μg/mL (*P. aeruginosa* ATCC 25923)	>1112 μg/mL				
14 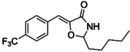	16 μg/mL (*S.aureus* ATCC 29213)		256 μg/mL		–	[137]
	4 μg/mL (MRSA ATCC BAA‐4A)		256 μg/mL		–	
15 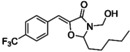	4 μg/mL (*S. aureus* ATCC 29213)		256 μg/mL		–	[137]
	4 μg/mL (MRSA ATCC BAA‐4A)		256 μg/mL		–	
JJM‐ox‐3‐70 (16) 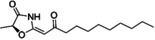	>128 μg/mL (*Salmonella Typhimurium*)	inhibition at >64 μg/mL			–	[138]
17 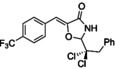	6 μg/mL (MRSA ATCC BAA‐44)	5 μM: 82% biofilm inhibition and 57% dispersal 40 μM: 69% dispersal			–	[139]

[a] X‐fold enhancement in assay measurement compared to linezolid control from the same study, if measured. [b] Study reported data for MRSA and MSSA as one figure under *S. aureus*. [c] Study used safranin to stain biofilm instead of crystal violet.

Methicillin resistant *Staphylococcus aureus* (MRSA) is associated with significant morbidity and mortality.^[113]^ Becerra and colleagues^[114]^ investigated the anti‐biofilm activity of linezolid against various strains of *S. aureus* (MSSA ATCC 29213, MRSA ATCC 43300, clinical strains MRSA 1 and MRSA 2). The efficacy of linezolid on these biofilms was determined using CV staining and XTT assays. CV established that at sub‐MIC concentrations (<1 μg/mL for MRSA ATCC 4300 and <2 μg/mL for MSSA ATCC 29213, MRSA 1 and MRSA 2), linezolid inhibited the reference strains MRSA ATCC 4300 and MSSA ATCC 29213 biofilms by 29.2% and 29.4% respectively in comparison to the control. When treating the clinical strains it performed significantly better with MRSA 1 and MRSA 2 achieving a 51.6% and 77.3% inhibition respectively. Linezolid's ability to eradicate mature biofilms was also established at 10x MIC and the biomass reduction was reported to be between 13–57%, with clinical isolates on the higher end of the range. The XTT assay also revealed that linezolid could eradicate the clinical strains (MRSA 1 76% and MRSA 2 51%) to a better extent than the reference strains (MRSA ATCC 43300 60.8% and MSSA ATCC 29213 32%) after 24 hours of treatment. SEM imaging of MRSA 1 and MRSA ATCC 43300 after 24 hours of treatment revealed 99.8% and 98.6% eradication respectively. It is important to note that Becerra and colleagues reported that the reference strains produced loosely attached biofilms and the clinical isolate biofilms were more strongly adhered to the surface. In this light, it is rather interesting that linezolid had better anti‐biofilm activity against the clinical isolates, and this work suggests that linezolid may play a role in disrupting the adhesion of bacteria in the biofilm, a crucial step in biofilm formation (section 1.1). This work also highlights the difference between using clinical and reference strains, and the importance of choosing appropriate strains for testing.

### 3.2 Tedizolid

Tedizolid (Figure 3) is a second generation oxazolidinone developed by Cubist Pharmaceuticals.^[115]^ It was approved in 2014 for the treatment of skin and skin structure infections.^[116]^ Tedizolid is marketed in its prodrug form tedizolid phosphate (Figure 3) (trade name Sivextro). The incorporation of the phosphate group improves its bioavailability and water solubility. In the body the phosphate ester is converted to the active compound by plasma or intestinal phosphatases.[^115^, ^117^]

Tedizolid extends the linezolid scaffold beyond the C‐ring through the inclusion of a biaryl system at the 4‐position of the 3‐fluorophenyl group. The morpholino group has been replaced with a pyridine moiety and a methyl tetrazole has been incorporated in the D‐ring position. Simultaneously, the C5 side chain has also been modified, and the 5‐acetamidomethyl moiety has been replaced with a hydroxymethyl group. Lupton, Belousoff and co‐workers^[105]^ have examined the binding region for linezolid and tedizolid, and both compounds adopt a similar binding pose. The inclusion of a biaryl ring system in tedizolid enables the formation of favourable hydrogen bonds within the binding pocket which leads to better potency in terms of MIC.

Bone and joint infections are extremely difficult to treat, with surgical intervention and long courses of antibiotic treatment resulting in failure.[^118^, ^119^] The anti‐biofilm capabilities of tedizolid against bone and joint infections has been evaluated by Laurent and colleagues (Table 1).^[120]^ It is important to note that most musculoskeletal infections are biofilm related with biofilms forming on bone, nearby implants, or both.^[121]^ The ability of linezolid and tedizolid to prevent biofilm formation in *S. aureus* (MSSA 6850 and 2 clinical MSSA isolates) was investigated. Both compounds were able to inhibit the biofilm formation of isolates tested. The biofilm MICs (bMIC) for tedizolid were reported to be 0.25 mg/L (MSSA 6850 and clinical MSSA isolate 1) and 0.5 mg/L (clinical MSSA isolate 2), and 1 mg/L (MSSA 6850 and clinical MSSA isolate 1) and 2 mg/L (clinical MSSA isolate 2) for linezolid. This makes tedizolid 4 times more potent than linezolid at inhibiting biofilm formation in *S. aureus*. Interestingly the bMICs for both compounds in MSSA 6850 and clinical MSSA isolate 1 are lower than their planktonic MICs. It is suspected that the explanation for this lies in the mechanism of action of oxazolidinones (section 3.2). As protein inhibitors, oxazolidinones may be able to inhibit the proteins involved in bacterial adhesion which is the first stage in biofilm formation (Figure 1). Therefore, this result highlights the possibility for these compounds to be incorporated into drug delivery systems such as bone cements or coated implants as a preventative measure (section 7). The effect of the compounds against mature biofilms was also investigated using an MBEC assay. Laurent and colleagues reported the MBEC values as >675 μg/mL and >2000 μg/mL for tedizolid and linezolid respectively. Although the reported MBEC for tedizolid is 3‐fold lower than for linezolid, it was reported that even with increasing concentrations of both compounds there was no significant difference to the bacterial inoculum and CFU counts hovered around 7 Log_10_ CFU/mL. Therefore, it was determined that both oxazolidinones alone are not effective at treating chronic forms of bone and joint infections as they were inactive against mature MSSA biofilms.

Infective endocarditis often occurs in health care settings, and *Enterococcus faecalis* accounts for approximately 97% of infective endocarditis cases.[^119^, ^122^] Zhou, Shen and colleagues investigated the activity of tedizolid and linezolid against linezolid‐resistant *Enterococcus faecalis* strains (Table 1).^[123]^ Using a crystal violet assay, they demonstrated that at 8 times the MIC linezolid performed slightly better than tedizolid at eradicating 6 out of 8 clinical isolates of linezolid‐resistant *Enterococcus faecalis*. Both compounds performed significantly better at inhibiting biofilms at sub minimum inhibitory concentrations (1/4× MIC and 1/8× MIC), with tedizolid having a greater effect. Therefore, tedizolid is more effective at inhibiting biofilm formation than eradicating established biofilms. It is suspected that the structural differences between the compounds results in different down regulation of biofilm related genes. However, more work is required to confirm this hypothesis.

Skin and soft tissue infections are extremely common worldwide but remain clinically challenging in terms of treatment.^[124]^ Lang and co‐workers^[82]^ investigated the *in vitro* efficacy of tedizolid against the sensitive and resistant *Staphylococci* often present in these infections (Table 1). The susceptibility of a range of *S. epidermidis* (clinical isolates including linezolid sensitive and resistant strains) and *S. aureus* (clinical MSSA and MRSA isolates including linezolid sensitive and resistant strains) biofilms was determined using an XTT assay with a resazurin stain. At 1× MIC, both antibiotics were unable to reduce the mean number of viable cells to below ≈70%. Linezolid performed slightly better than tedizolid against *S. aureus* strains (64% versus ≈70% mean cell survival). The reverse was seen for *S. epidermidis* where tedizolid (≈85% mean cell survival) did slightly better than linezolid (103% mean cell survival). At 10× MIC, tedizolid was able to significantly reduce the viable cells of both *S. aureus* and *S. epidermidis* strains (≈30% and 35% mean cell survival). This makes tedizolid approximately 1.5 times more potent than linezolid against *S. aureus* and 2.3 times more potent against *S. epidermidis*. A flow‐cell system was also used in the study to replicate *in vivo* conditions. Under flow‐cell conditions at 10× MIC, both antibiotics were comparable, and they were able to reduce the number of viable cells substantially (8%, p<0.05 cell survival for tedizolid and 12%, p<0.005 cell survival for linezolid) when compared to the untreated biofilm control. These results also highlight the importance of selecting the appropriate biofilm testing assay as different methods can provide different conclusions (refer to discussion in section 2).

## 4 Biofilm activity of structural variants of linezolid

**Figure 4:**
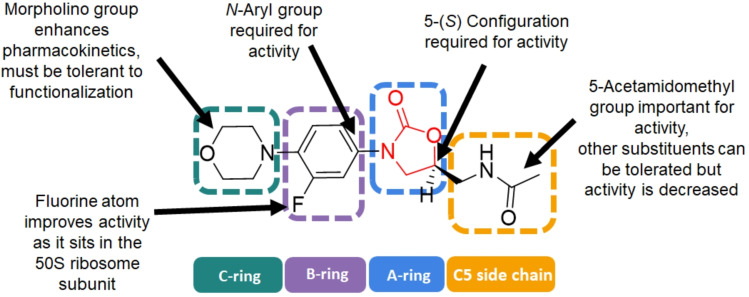
The chemical structure of linezolid (1) and its structure‐activity relationships.

The pharmacophore of linezolid (Figure 4) consists of the oxazolidinone ring (A‐ring), the 5‐acetamidomethyl group (C‐5 side chain), the 3‐fluorophenyl ring (B‐ring) and the morpholine unit (C‐ring). The stereochemistry at the 5‐position in the oxazolidinone ring is important for activity and must be in the *S* configuration as the *R* configuration has no antimicrobial activity.^[125]^ The *N*‐aryl group has been determined to be required for activity and many analogues leave the B‐ring unchanged for potent antimicrobial activity. The 5‐acetamidomethyl group is also generally left unchanged in analogues to maintain potent activity. While small substituents such as hydroxyl groups can be tolerated, larger substituents such as phenyl groups result in a loss of activity. In prior attempts to replace the side chain with various heterocycles, all resulting analogues had either reduced or no antimicrobial activity (*S. aureus* strains ATCC 9144, NCTC 13616, USA 300 and 1199B; *E. faecalis* strains VSE NCTC 775 and VRE NCTC 12201, the *E. faecium* strain VRE NCTC 12204, *K. pneumoniae* strains NCTC 13368 and M6; *A. baumannii* strains AYE and ATCC 17978; *P. aeruginosa* strains PA01 and NCTC 13437; *E. coli* strain NCTC 12923).^[126]^ However activity in a different group of bacterial strains (*M. tuberculosis* ATCC 27294, MRSA strain SAU1009, a range of MRSA, MRSE, MSSE, *Staphylococcus hemolyticus, Staphylococcus saprophyticus, Streptococcus pyogenes, Streptococcus agalactiae, Streptococcus mitis* strains) was shown to increase when the morpholino unit is also simultaneously modified with a 5‐acetamidomethyl group as seen in the oxazolidinone contezolid (where the morpholino group is replaced with 2,3‐dihydro‐1*H*‐pyridin‐4‐one and the 5‐acetamidomethyl moiety is replaced with *N*‐methyl‐1,2‐oxazol‐3‐amine)^[60]^ and posizolid (which also incorporates *N*‐methyl‐1,2‐oxazol‐3‐amine as the C5 side chain but replaces the morpholino unit with (*S*)‐1‐(3,6‐dihydropyridin‐1(2*H*)‐yl)‐2,3‐dihydroxypropan‐1‐one).^[127]^ The morpholino moiety was found to improve the water solubility and pharmacokinetics of the drug.^[102]^ The 4‐position of the morpholino group is the most tolerant to functionalisation and efforts to synthesise more potent oxazolidinones will often have analogues with modifications at this position. In the literature the morpholine unit has been replaced with a number of different groups without a significant loss in activity (MRSA ATCC 33591, MRSA ATCC 43300, MRSA H‐29, MSSA ATCC 29213, MSSA ATCC 25923, MSSA ATCC 19636, MSSA ATCC 6538, MRSE 35984, *E. faecalis* 427, and *S. pneumoniae* 5051.[^128^, ^129^, ^130^]

### 4.1 Modification of the morpholine unit: biaryl ring systems

**Figure 5:**
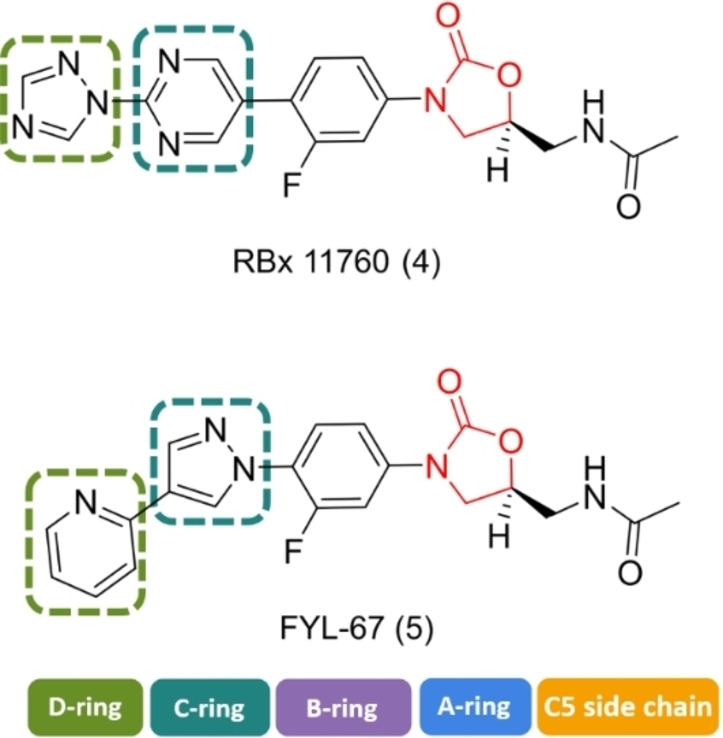
Chemical structures of RBx 11760 (4) and FYL 67 (5) which include biaryl ring systems.

A number of oxazolidinones have been developed based on structural modifications to the morpholine group of the linezolid scaffold. Ranbaxy Research Laboratories reported the synthesis of three biaryl oxazolidinones, but only RBx 11760 (Figure 5) was tested in biofilms (Table 1).[^130^, ^132^] RBx 11760 possesses a 1,2,4 triazole in the D‐ring position and a pyrimidine in the C‐ring position.^[132]^ Using a crystal violet staining assay, it was demonstrated that RBx 11760 was able to inhibit the formation of methicillin resistant *S. epidermidis* (MRSE) (ATCC 35984) biofilms. At 16× MIC (4–8 μg/mL) a 95% inhibition was achieved making RBx 11760 approximately 4–8 fold more potent than linezolid for this strain. A plate count assay was used to investigate the biofilm eradication ability of RBx 11760 *in vivo* on MRSA H‐29 biofilms in mouse models. RBx 11760 was more effective in treating skin and soft tissue infections, requiring half the dose of that required by linezolid. To investigate the eradication ability further, a foreign‐body mouse biofilm infection model (where MRSE ATCC 35984 and *S. aureus* Xen‐29 biofilms were grown on implanted catheters) was devised. Mice were treated with either linezolid or RBx 11760 at a dose of 40 mg/kg twice a day, and over 3 days, RBx 11760 demonstrated significant anti‐biofilm potency whereas linezolid resulted in no improvement. These results suggest that the replacement of the morpholino group in linezolid with the biaryl ring system seen in RBx11760 may be important for increasing the potency of oxazolidinones against biofilms.

The biaryl oxazolidinone FYL‐67 (Figure 5) developed by Wang and colleagues^[129]^ consisted of a pyrazole unit in the C‐ring position and a pyridine moiety in the D‐position. Wang and co‐workers demonstrated that FYL‐67 had anti‐biofilm activity against a range of *S. aureus* strains (methicillin susceptible *S. aureus* ATCC 25923, MRSA ATCC 33591, MRSA ATCC 43300 and clinical MRSA isolates) (Table 1).^[134]^ In the study, MBC and MBEC assays were used to determine the anti‐biofilm activity of FYL‐67 by measuring biofilm inhibition using colony counting. The MBEC assays showed that FYL‐67 was able to eradicate methicillin susceptible *S. aureus* and MRSA biofilms at concentrations of 256 μg/mL and 128 μg/mL respectively making the oxazolidinone analogue two times more potent than linezolid. The MBC assays corroborated this finding for all strains except MRSA ATCC 33591 where FYL‐67 and linezolid were shown to be equipotent. The effect of the compound on biofilm formation was also investigated. Wang and colleagues used a modified microtiter assay^[140]^ and analysed the biofilms using crystal violet and resazurin staining. At 0.5x MIC FYL‐67 exhibited better potency against the inhibition of mature biofilms for all MRSA and MSSA strains (42.7–52.1%). In contrast linezolid only had a modest effect on the inhibition of the mature biofilms for MSSA ATCC 25923 (25% compared to 52.1% for FYL‐67). Although FYL‐67 performed better than linezolid in the inhibition of mature biofilms, both compounds were similarly not as effective at inhibiting young biofilms at the same concentrations (20–35% for both compounds).

**Figure 6:**
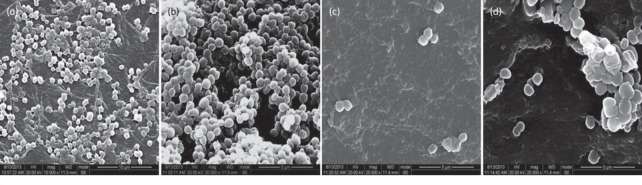
SEM images of *S. aureus* biofilms formed *in vivo on catheters*. a) *S. aureus* ATCC 25923 biofilms before treatment with FYL 67 (5), b) *S. aureus* ATCC 25923 biofilms before treatment with linezolid (1), c) eradication of biofilm after treatment with FYL 67 and d) after treatment with linezolid (1). Reproduced with permission. Ref. [134] Copyright 2014, Oxford Academic.

The *in vivo* efficacy of FYL‐67 was investigated using a mouse model of a catheter‐associated infection from *S. aureus* ATCC 25923 biofilms and the catheters were examined using scanning electron microscopy (SEM) imaging (Figure 6) to obtain viable cell count estimates. The mice in the study received a daily dose (10 mg/kg) of either linezolid or FYL‐67. When compared to linezolid, the number of viable bacteria on the catheter was 10^3^ to 10^4^ times lower for FYL‐67. The reduction of viable bacteria on the skin was also significant and the CFU counts obtained for FLY‐67 were 10^10^ to 10^7^ times lower than that of linezolid. These observations suggest that FYL‐67 is not only able to eradicate catheter associated biofilm infections but also bacteria in close proximity on the skin. While the exact mechanism of action for biofilm eradication or dispersal is unknown, the findings from this work suggest that FYL‐67 prevents the maturation of *S. aureus* biofilms due to the inhibition of protein and carbohydrate production in the EPS. Considering that the formation of the EPS is a crucial step in biofilm formation,[^22^, ^23^] this result may help to further elucidate the mechanism of action of FYL‐67 in biofilms.

### 4.2 Modification of the morpholine unit: aryl ring systems with linkers

**Figure 7:**
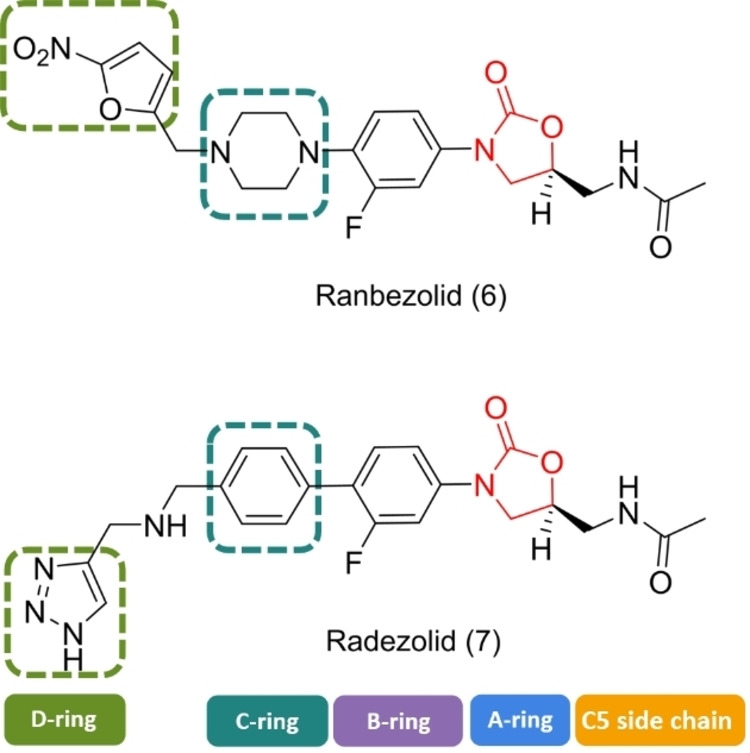
Chemical structures of ranbezolid (6) and radezolid (7).

Ranbezolid (6) also known as RBx 7644 extends the linezolid scaffold beyond the D‐ring position (Figure 7). Ranbezolid possesses a piperazine moiety in the C‐ring position linked to a nitrofuran by way of a methylene linker. Ranbezolid is well known for *in vitro* activity against gram‐positive pathogens, especially methicillin‐susceptible *S. aureus* and MRSA, as well as *S. epidermidis* and MRSE (Table 1). In addition, it showed antibacterial efficiency against gram‐negative pathogens, gram‐positive anaerobes, and slime‐producing *Staphylococci* and *Mycobacteria*.[^141^, ^142^] Raj and co‐workers studied the interaction of ranbezolid with the cell wall and ribosomes. They investigated the membrane integrity of *Staphylococci* (*S. aureus* and *S. epidermidis*) and the inhibition of the *in vitro* translation system by ranbezolid compared to linezolid at different concentrations by using a SYTO 9/PI assay. Ranbezolid and linezolid had no effect on the membrane integrity at 4 μg/mL on *S. aureus*. Interestingly, they found membrane‐damaging activity on *S. epidermidis* at 2× MIC (2 μg/mL) but no effect on the membrane at 1× MIC (1 μg/mL), similar to membrane‐disrupting drugs valinomycin and carbonyl cyanide *m*‐chlorophenylhydrazone. Raj and co‐workers suggested the nitrofuran ring may be responsible for the damage of the membrane integrity in *S. epidermidis*. The role of ranbezolid as an inhibitor of bacterial ribosomes was examined by an *in vitro* cell‐free transcriptional and translation assay (S30 and TnT assay). The IC_50_ of ranbezolid was 17 μM, demonstrating 5–6 fold better inhibition than linezolid. The data also showed that ranbezolid is a potent bacterial protein synthesis inhibitor and does not inhibit mammalian protein synthesis. In further docking studies, various conformations of ranbezolid and their interactions with the 50S ribosome were studied. It is likely that ranbezolid and linezolid have similar interactions with ribosomes, however ranbezolid can build additional van der Waals and H‐bonding interactions due to the nitrofuran moiety and therefore is a better inhibitor.

In a study by Rattan and colleagues, the effect of different antibiotics on biofilms was tested.^[136]^ Biofilms were grown with the slime producing isolates MRSE 879, MRSA 562, MRSA 1026/99, and *S. epidermidis* ATCC 35983, determined via OD_544nm_ measurements. The data showed that ranbezolid significantly inhibited biofilm formation, more efficiently than vancomycin, quinupristin/dalfopristin, or linezolid against both *S. aureus* (MRSA 562, 1026/99) and CNS (coagulase‐negative *Staphylococci*) and inhibits biofilm formation at sub‐MIC and MIC concentrations (MRSE 879 0.5 μg/mL, MRSA 562 1 μg/mL, MRSA 1026/99 2 μg/mL, *S. epidermidis* ATCC 35983 0.25 μg/mL). In a follow‐up study, the authors studied the time‐dependent anti‐adhesive effect of ranbezolid and other antibiotics on polystyrene surfaces.^[133]^ The results show that ranbezolid has anti‐adhesion potential as this drug was able to significantly decrease the activity against adhesion and therefore prevent biofilm growth of staphylococcal cells (*S. aureus* and *S. epidermidis*) even with a 2 to 6 hour delay. The literature supports the potency of ranbezolid as an antibacterial drug against *Staphylococcus* infection and an inhibitor for biofilm formation, providing advantages over linezolid.

Radezolid (RX‐1741) (Table 1) consists of a benzene ring in the C‐ring position connected to a 1,2,3‐triazole via a dimethylamine linker. This broad‐spectrum antibiotic shows excellent *in vivo* antibacterial activity against gram‐positive bacteria such as *Staphylococci* (MIC_90_ 1–4 μg/mL), and *Enterococci* (MIC_90_ 0.5–1 μg/mL).^[143]^ Radezolid (7) also has good activity against some gram‐negative bacteria, such as *Haemophilus influenzae* and *Moraxella catarrhalis*, with MIC_90_ values of 1 μg/mL and 0.5 μg/mL, respectively (Figure 7).^[144]^ The high potency of the drug is explained by the additional interaction of the tetrazole ring with the binding pocket of the target 50S ribosomal subunit.[^144^, ^145^]

In a recent study, Qu and colleagues investigated the effect of radezolid on planktonic and biofilm cells of *Enterococcus faecalis* and compared the results with linezolid.^[146]^ The authors found a significantly greater effect of radezolid on planktonic *E. faecalis* cells than linezolid. The biofilm biomass was evaluated by crystal violet staining and the adherent cells were quantified according to CFU numbers. Interestingly, the results on eradication of already established biofilms or adherent cells were the same for both drugs. However, radezolid was more effective at inhibiting biofilm formation (drugs were used at 1/4–1/8× their MICs on isolates 16C106 and 16C350) than linezolid. The authors showed results which indicated that the transcription of some genes (*ahrC*, *esp*, *relA*, *relQ*) in *E. faecalis* is effectively inhibited by radezolid. In another recent study, Shen and co‐workers investigated the effect of linezolid, tedizolid, and radezolid on different linezolid‐resistant *E. faecalis* clinical isolates.^[123]^ Radezolid showed the best efficiency to eradicate biofilms (1/8x MIC) for most strains (except FC2021 and FC2471), followed by linezolid and tedizolid. However, tedizolid (1/4× MIC) was more effective at inhibiting biofilm growth than radezolid (1/8× MIC) and linezolid (1/16× MIC). As shown here, radezolid shows promising results as an effective oxazolidinone with stronger potency against linezolid‐resistant strains.^[147]^

## 5 Anti‐biofilm properties of other 2‐oxazolidinones

**Figure 8:**
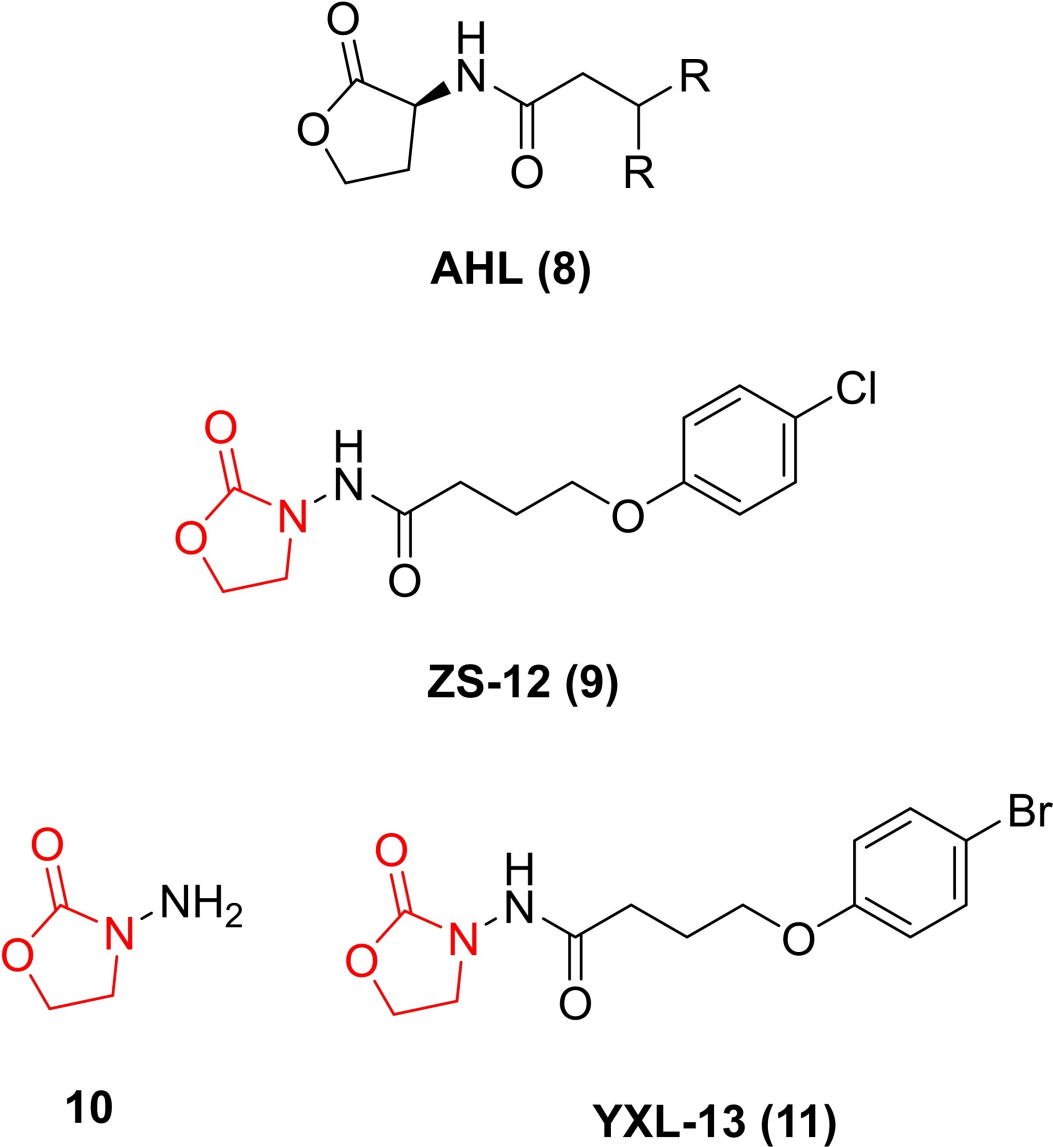
The chemical structure of an AHL (8), a quorum sensing signalling molecule (9), a bioisostere of an AHL (10) and the analogue YXL‐13 (11).

A number of oxazolidinones based on scaffolds other than linezolid have been reported, but very few have been tested against biofilms. AHL molecules (such as (8) – Figure 8), have been studied extensively as AHL‐dependent quorum sensing (section 1.1) is essential for the pathogenicity of *P. aeruginosa*.^[16]^ However, under basic conditions, the lactone ring of AHLs can easily undergo ring opening and activity is lost. To circumvent this issue, Lin and co‐workers reported the synthesis of a series of 3‐amino‐2‐oxazolidinone derivatives (such as lead compound 9), as bioisosteres (10) for the AHL lactone, with activity against *P. aeruginosa*.^[17]^ It is unknown whether these compounds also target the 50S ribosome as they were designed to modulate QS. The oxazolidinone ring was found to be essential to the antimicrobial activity and was retained in the series of oxazolidinone compounds synthesised. The compounds were initially evaluated against *C. violaceum*.^[148]^ The key structural features required for anti‐QS activity was found to be a short alkyl chain between the 2‐oxazolidinone and benzene ring, and a weak electron withdrawing group in the *para* position on the benzene ring. The best compound was found to be YXL‐13 and it was evaluated against *P. aeruginosa* biofilms. Using a crystal violet assay, YXL‐13 was found to inhibit the formation of *P. aeruginosa* PAO1 biofilms by 40.39% at 162.5 μM. YXL‐13 was also shown to inhibit four virulence factors (pyocyanin, elastase, rhamnolipid and protease) involved in the pathogenesis of *P. aeruginosa* and regulated by QS. Lin and colleagues demonstrated that YXL‐13 also works synergistically with antibiotics such as meropenem trihydrate to increase their susceptibility to biofilm cells. Although further testing is required, these preliminary results highlight the potential of improving the potency of oxazolidinones against gram‐negative bacteria.

## 6 4‐Oxazolidinones with anti‐biofilm activity

**Figure 9:**
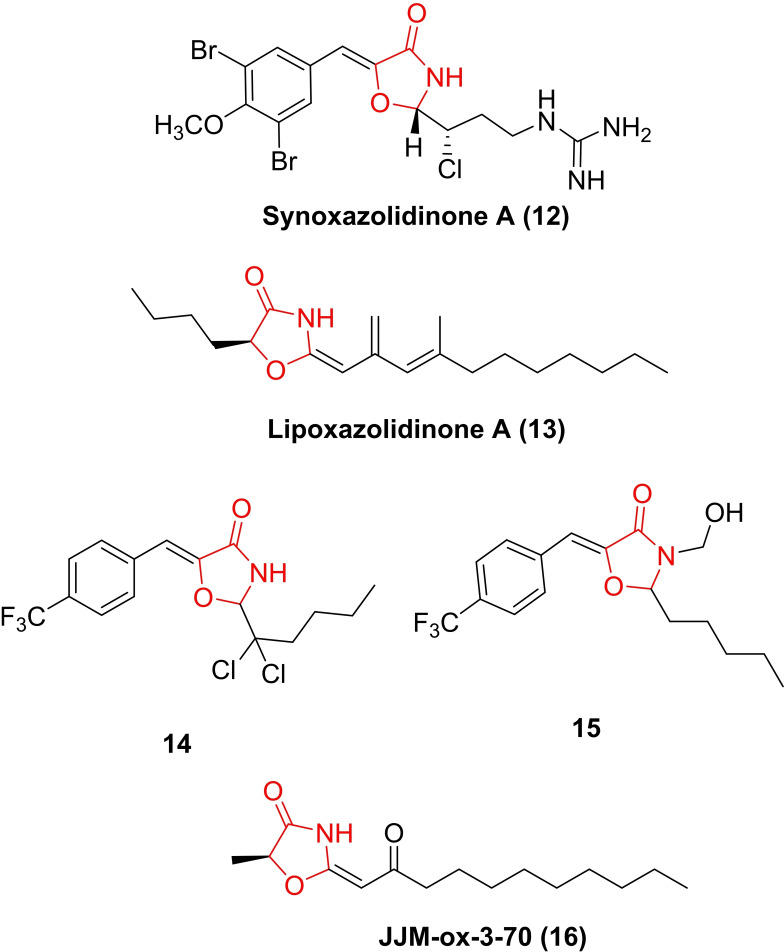
Biologically active 4‐oxazolidinones found in nature: synoxazolidinone A (12) and lipoxazolidinone A (13), along with analogues 14, 15 and JJM‐ox‐3‐70 (16) which display antibiofilm activity.

The occurrence of 4‐oxazolidinones in nature is rare, with only 2 classes reported: the lipoxazolidinones (isolated from marine actinomucetes from a Guam marine sediment)^[149]^ and the synoxazolidinones (isolated from the sub‐arctic ascidian Synoicum pulmonaria).^[150]^ While the exact mechanism of action is unknown, both the lipoxazolidinones and synoxazolidinones and their analogues[^151^, ^152^] have antimicrobial properties, with the oxazolidinone ring found to be essential for antibacterial activity. Synoxazolidinone A possesses an unusual 5‐benzylidene‐4‐oxazolidinone core decorated with a brominated aromatic moiety and chlorinated, guanidine‐containing side‐chain. Synoxazolidinone A and its related natural product, synoxazolidinone C,^[153]^ have been shown to exhibit anti‐fouling properties against a variety of marine bacteria.^[154]^ Pierce and colleagues[^139^, ^155^] identified 2‐dichloroalkyl‐5‐benzylidene‐4‐oxazolidinones (17) (Table 1) as modulators of MRSA biofilms (ATCC BAA 44). Through preparation of a series of analogues, key structural features were identified for anti‐biofilm potency (measured as biofilm inhibition using the established crystal violet staining protocol).^[156]^ These key moieties include the 4‐oxazolidinone core, a small to medium alkyl chain on the aminal carbon of the 4‐oxazolidinone, electron‐withdrawing groups on the benzylidene moiety, and the presence of the dichloroethylene functionality were critical to activity. The current lead compound (14) is shown alongside lipoxazolidinone A (13) and synoxazolidinone A (12) in Figure 9.

With the ability of synoxazolidinone natural product analogues (5‐benzylidene‐4‐oxazolidinones) to disperse biofilms established, Pierce and co‐workers^[137]^ demonstrated that related analogues, such as the formaldehyde hemiaminal of the oxazolidine core 15, work synergistically with antibiotics such as doxycycline (reducing the antibiotic's MBEC) to eradicate pre‐formed *S. aureus* ATCC 29213 biofilms. Subsequently, Pierce and colleagues^[138]^ examined the activity of the 4‐oxazolidinone analogue JJM‐ox‐3‐70 (16) against the gram‐negative pathogen *Salmonella typhimurium*. JJM‐ox‐3‐70 was shown to inhibit biofilm formation by decreasing virulence through disruption of biofilm matrix gene expression (specifically promoters for curli and the flagellar filament) and altering swimming motility.

## 7 Oxazolidinone delivery systems targeting biofilms

The key role of biofilms in enhancing bacterial pathogenicity, increasing severity of infection and potentially chronic infections has been introduced earlier (section 1.1). Biofilms are strongly associated with in‐dwelling devices and medical implants,[^157^, ^158^] chronic wounds,^[159]^ and patients suffering from pulmonary diseases.[^158^, ^160^] Therefore, targeting effective antibiotics, like oxazolidinones, to these common points of infection offers the potential to greatly enhance their efficacy and overcome the resistance mechanisms of biofilms.^[161]^ Owing to the short development history of oxazolidinones, and their use as last resort antibiotics, there has not been a strong demand for delivery systems, with most focused on linezolid. But with the continuing development of antibiotic resistance, delivery systems that can target oxazolidinones to bacterial biofilms will see an increase in demand. Here we will review some delivery systems that target common sources of bacterial biofilm infection and have been tested against biofilms or in relevant preclinical models.

### 7.1 Microparticle and nanoparticle delivery agents

Microparticles and nanoparticles have been developed as delivery vectors for oxazolidinones, to improve solubility and bioavailability, to enhance uptake into bacterial biofilms, to target intracellular biofilms, or to control release. It is the specific physicochemical properties of particle systems that improve these outcomes, targeting of biofilms or tissues to enhance localised concentrations, intracellular extravasation of nanosized particles or enhanced pharmacokinetic profiles.

**Figure 10:**
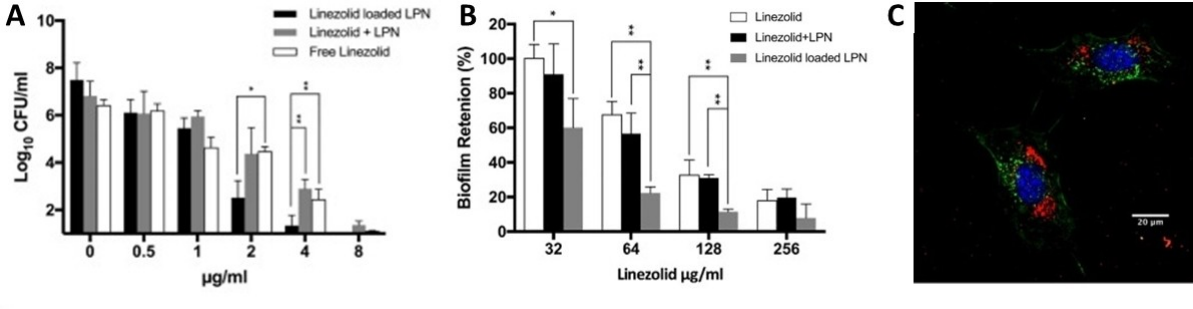
Linezolid loaded polymer nanoparticles (LPN) can treat biofilms and intracellular biofilms associated with osteomyelitis infections. (A) Eradication of intracellular MRSA (USA3000114) biofilms grown in osteoblasts (MC3T3E1) is enhanced by loading the nanoparticles with linezolid. (B) Linezolid loaded LPNs are effective against MRSA biofilms grown in a microplate assay, biofilm retention determined by CV assay. (C) Confocal laser scanning microscopy images of osteoblast cells (nuclei: blue, membranes: green) treated with linezolid LPNs (red). Adapted with permission. Ref. [135] Copyright 2020, Elsevier.

Osteomyelitis is a challenging infection of the bone, commonly caused by *Staphylococci* strains. Difficulty in treating this disease arises from the limited penetration of many antibiotics into bone, as well as the ability of *Staphylococci* infections to protect themselves as biofilms or intracellular colonies.^[162]^ To address this issue, Wong and colleagues^[135]^ synthesised linezolid loaded lipid‐polymer hybrid nanoparticles to target bone tissue. These nanoparticles are capable of increasing the localised concentration of linezolid in bone and targeting intracellular biofilms in osteoblasts. The hybrid particles consisted of linezolid loaded into a poly(lactide‐co‐glycolide) (PLGA) core, encapsulated in a lipid coat of lecithin, pegylated‐phospholipids and cholesterol. The particles showed a maximum loading of 12 wt% linezolid, and a biphasic release, with 25% being released in the first 12 hours, but a sustained release up to levels greater than 70% over 120 hours. The drug release was slower in lipid coated nanoparticles, compared to PLGA nanoparticles, demonstrating that the lipid provided a barrier against burst release of linezolid. While the linezolid‐nanoparticles demonstrated a higher MIC (approximately two‐fold greater) in planktonic assays compared to linezolid, they demonstrated much better efficacy against intracellular colonies in osteoblasts (12–87 fold increase in efficacy) and against microtiter biofilm assays (1.5–3 fold increase, Figure 10A, B). The internalisation of the nanoparticle into viable osteoblasts was demonstrated using confocal microscopy (Figure 10C) and cell viability assays. In a first step towards expanding beyond *in vitro* evaluation, it was shown in a rat model that bone tissue concentration of linezolid could be increased by over 400% by incorporation into the lipid hybrid nanoparticles. This offers an exciting opportunity to develop novel therapies for osteomyelitis, by targeting bone tissue, biofilms, and intracellular colonies of bacteria.

Oxazolidinones as a class of antibiotics are commonly used against gram‐positive bacteria, with a lower efficacy against gram‐negative strains. While resistance is not a common problem for oxazolidinones, this is an inevitable challenge that will need to be addressed. One capability of nanoparticle delivery is the potentiation of oxazolidinone activity against less susceptible strains. Recently, Eltayeb and co‐workers^[163]^ investigated a subset of strong biofilm forming isolates from a library of clinical samples, predominantly of *S. aureus* and *S. epidermidis*. All strong biofilm forming isolates were resistant against linezolid (MIC ≥8 μg/mL), doxycycline (MIC ≥16 μg/mL) and clindamycin (MIC ≥4 μg/mL). These antibiotics were reformulated into lipid nanoemulsions, with a size range of 10–15 nm and a zeta potential between −9 to −15 mV, and then retested for efficacy against biofilms in a minimum biofilm inhibitory concentration (MBIC) assay. For linezolid it was found that 87% of *S. aureus* and 57% of *S. epidermidis* biofilms were sensitive to the nanobiotics, which was much higher than for doxycycline and clindamycin. The potentiation of linezolid was assigned to improved bioavailability of the lipophilic drug, which had similarly been seen for oral bioavailability of microemulsions of linezolid and two other oxazolidinones in rabbits.^[164]^ What wasn't considered in this report was the small size, and negative surface charge of the nanoemulsions, which could also be influencing biofilm or bacterial cell penetration. Another recent example by Sinicropi and co‐workers^[165]^ demonstrated potentiation of simple allyl and aryl oxazolidinone analogues against gram‐negative bacteria by incorporation into nanoparticles. Three different nanoparticle formulations were investigated: methacrylic acid grafted poly(*N*‐vinyl‐pyrrolidone), precipitation polymerisation of methacrylic acid crosslinked with ethylene glycol dimethacrylate, and a biobased nano‐emulsion of soybean lecithin. In all cases the nanoparticle formulations demonstrated an increase in efficacy against the gram‐negative strains *E. coli* and *S. cerevisiae* (MIC 4 μg/mL), compared to the free drugs (MIC 16 μg/mL). While this report demonstrates that nanoparticles can potentiate efficacy of oxazolidinones against gram‐negative bacteria, the characterisation of the three particle classes was limited, so does not provide insight into the mechanism of action. While using nanoparticles to potentiate the activity of oxazolidinones against non‐susceptible bacterial biofilms, a more thorough investigation of the physicochemical properties of the nanoparticles, and how this influences biofilm and cellular penetration is necessary to fully elucidate the mechanism of action.

Owing to the importance of the biofilm phenotype in chronic pulmonary infections such as tuberculosis and the *P. aeruginosa* infections commonly associated with cystic fibrosis, efficiently targeting antibiotics to the lungs is critical. There have been recent examples investigating the design of linezolid nanoparticles,^[166]^ and composite nanoparticle‐microparticles,^[167]^ for inhalation therapy. While both these reports demonstrate that careful material design can lead to improved drug encapsulation, sustained release, and good biocompatibility with host cells, they have not yet evaluated their efficacy against bacterial biofilms. While linezolid has been the most widely studied oxazolidine for delivery by nanoparticles, a recent example by Luo and colleagues^[168]^ has created polymeric micelles loaded with FYL‐67 (pyrinezolid) (5) (Table 1). Biodistribution studies showed increased drug concentration in lung tissues of mice treated with the micelles intravenously, compared to the free drug alone. This was hypothesised to relate to structure and physiology of lung tissues, which is beneficial to nanoparticle enrichment in these tissues. Rats infected systemically with MRSA and treated with the FYL‐67 micelles showed a greater survivability, compared to free drug, owing to the improved pharmacokinetic and biodistribution profiles.^[168]^ While this was proposed to demonstrate the potential of the FYL‐67 micelles to fight MRSA related pneumonia, no evaluation against a relevant model has been reported yet.

### 7.2 Bone cements and fillers

Osteomyelitis is a severe bone infection commonly associated with commensal *Staphylococci*, with drug resistance and biofilm formation being critical complicating factors leading to chronic disease.^[169]^ Disease management commonly involves debridement of infected and necrotic tissues, in combination with antibiotics, though effective treatment regimens are complicated by the limited number of clinical trials. Linezolid is an emerging therapeutic choice, owing to a lack or resistance due to its short history of clinical usage, as well as good bone tissue penetration. Bone cements, fillers and blocks, have been used in osteomyelitis treatment to improve healing of bone tissue, following debridement, debridement, and antibiotic loaded materials offer the potential of high concentrations of drug at the diseased tissue, potentially reducing side effects.^[170]^

A simple approach is the use of biodegradable microparticles encapsulated as spacers within poly(methyl methacrylate) bone cements, to control drug elution.^[171]^ Biodegradable poly(lactide‐co‐glycolide) was used by San Román and colleagues to form microparticles to co‐encapsulate vancomycin and linezolid.^[171]^ The therapeutics showed biphasic release profiles, with over 80% of linezolid and 20% of vancomycin released in 55 days. Despite the low elution of vancomycin, this formulation was shown to be most effective in antibiotic halo assays, showing that both drug synergy and concentration may be important factors in effective treatments.^[171]^ These biodegradable cements have been evaluated in a rabbit model for osteomyelitis, comparing single drug cements of vancomycin to linezolid.^[172]^ In this preliminary study PLGA‐antibiotic loaded bone cements showed decrease levels of infection, and improved tissue restructuring, compared to the cements loaded with the antibiotic directly. This preliminary histological study was not able to discern any significant differences between the two antibiotics in the PLGA cements, though it is worth noting the challenges due to a lack of effective preclinical models and standards for evaluation.

An alternative to the traditional, non‐biodegradable acrylic cements, are bioactive and osteoconductive bone fillers, such as calcium phosphates and apatites.^[173]^ Calcium deficient apatites loaded with linezolid have been investigated by Bouler and co‐workers^[174]^ for use as bone filling substitutes, following surgical debridement of osteomyelitis. Incorporation of 10 and 50 wt% linezolid was achievable, with 50 wt% loading showing enhanced porosity, and slower release kinetics (>95% release in 26 days, compared to 9 days). The linezolid‐CDA filler materials were tested against an osteomyelitis infection of MRSA in a rabbit model.^[175]^ It was found that intravenous linezolid and CDA‐linezolid material demonstrated statistically similar efficacy over 14 days, though there were only a small number of animals included in the study. Interestingly, there was a synergistic effect for the combined use of intravenous and CDA loaded linezolid. This suggests a potential therapeutic approach in humans of drug loaded bone filler material for localised and sustained drug concentration, with an early systemic delivery of linezolid to reduce bacterial levels.^[175]^

### 7.3 Coatings

Owing to the strong association of medical device related infections and biofilms,^[159]^ drug eluting coatings offer an opportunity to develop new protection against infection.^[161]^ A simple approach developed by Chhibber and colleagues^[176]^ is the encapsulation of linezolid within biodegradable poly(DL‐lactide). Coatings of varying concentrations of linezolid (2.5–10 wt%) could be applied to surgical wires through a simple dip coating process. All three coatings were able to sustain a localised concentration of linezolid greater than the MIC, over 120 hours. All three coatings demonstrated decreased levels of bacterial adherence in the first 48 hours, with drug loading of 5 and 10 wt% showing lower levels compared to 2.5 wt%, and three log orders lower attachment than uncoated or PDLLA only samples. Bacterial attachment to an implant surface is the critical first step in biofilm formation (section 1.1), so this provides exciting preliminary evidence of the ability of these materials to protect against biofilm infection.

**Figure 11:**
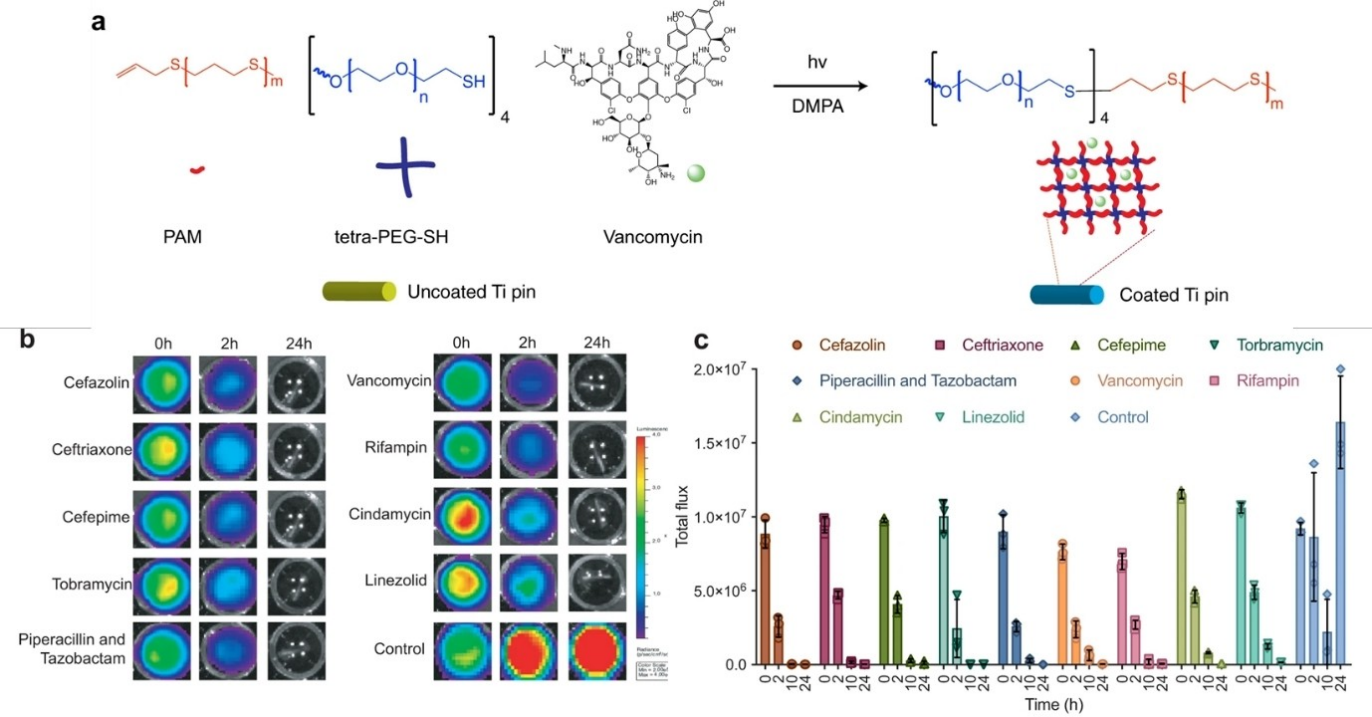
Point‐of‐care antibiotic coatings that can be applied using a simple process initiated by light. (A) Polyallyl mercaptan and four arm mercaptopolyethylene glycol stars were crosslinked using UV light and a photocatalyst to create antibiotic loaded coatings on medical devices such as titanium pins. (B) Efficacy of coatings on titanium pins, loaded with different antibiotics, was evaluated using a microtiter plate assay and a bioluminescent strain of *S. auereus*, with (C) quantified results over time. Adapted with permission.^[178]^ Creative Commons CC BY https://creativecommons.org/licenses/by/4.0/.

A challenge for coatings technologies is the necessity of regulatory approval of a new coating on an existing medical device. A new approach has been developed by the groups of Bernthal and Segura, that allows for biodegradable coatings to be applied at the point‐of‐care, independent of the implant being used. In this work, a network of polyethylene glycol and polyallyl mercaptan can be formed *in situ*, via a thiol‐ene ‘click’ reaction initiated by UV light.^[177]^ Similar networks were previously shown to encapsulate a range of drugs, and have good binding to metal surfaces, perfect for use with metal implants, but complex formulation was not amenable to point‐of‐care use.^[178]^ A range of common and last resort antibiotics (including linezolid) were encapsulated within these networks, coated onto titanium wires, and tested for their antibacterial and anti‐biofilm efficacy.^[177]^ All antibiotics tested showed a complete inhibition of *S. aureus* by 24 h, though linezolid was slower than other antibiotics tested (Figure 11). More in depth testing of the beneficial antimicrobial properties, drug release profiles and osseointegration of the materials loaded with vancomycin were performed. But, as there is increasing prevalence of vancomycin resistant *Staphylococci* infections in bone related disease, linezolid and oxazolidinones offer new opportunities that warrant further investigation in these materials.^[179]^

### 7.4 Biofabrication and advanced therapeutic systems

With the development of biofabrication techniques such as 3D printing, electrospinning, and 3D cell culture, new technologies with highly defined physical, chemical and biological properties, are being developed to treat diseases and support tissue healing.^[180]^ Electrospinning of biodegradable poly(DL‐lactic‐co‐glycolic) acid (PLGA) nanofibers loaded with linezolid have been investigated by Boncu, Guclu and collaborators for efficacy against MRSA in bone tissue applications.^[181]^ Through careful selection of solvent and electrospinning conditions, optimal fibre formulations could be produced that allowed for a loading of 13 wt% linezolid with 67% encapsulation efficiency, and showed sustained release up to 28 days, and *in vitro* inhibition of MRSA for up to 16 days. A follow up study, investigating more thoroughly the *in vitro* performance of these PLGA mats, as well as composite mats spun with polycaprolactone (PCL), found the same PLGA formulation was still the highest performing.^[182]^ This optimised formulation was tested against an *in vivo* tibial fracture model infected with MRSA. They compared treatment with systemic linezolid, prophylaxis with the mat (inserted during surgery) and treatment with the mat (inserted three days after initial surgery). Both the prophylaxis and treatment regimens showed enhanced therapeutic effect, compared to systemic treatment and no treatment. Interestingly the insertion of the mat three days post operation led to no detectable levels of MRSA at any of the time points investigated. Comparatively, the prophylactic group did not reach these same levels of bacteria load until day 14 of the study. These mats offer significant benefits in terms of decreasing the dose of antibiotic required for treatment and increasing patient compliance with only one intervention at time of surgery, therefore improving the efficacy and cost of treatment.^[182]^

As with other material delivery approaches, electrospun fibres and mats can allow for multiple therapeutic agents to be delivered, to enhance efficacy through synergy. Recently Miller and colleagues^[183]^ have developed conformable coatings, amenable to a variety of surfaces, that can encapsulate multiple antibiotics. The coatings comprised of composites of PLGA and PCL, allowing for tuning of the drug release profile depending on which polymer each drug was loaded into. Combinations of vancomycin, linezolid, daptomycin and rifampicin were investigated, and in all cases combination therapies were more efficacious than monotherapy. Composites of linezolid with rifampicin, and daptomycin with rifampicin, outperformed all other formulation in antibacterial efficacy and biocompatibility in both *in vitro* testing and an *in vivo* mouse model. The linezolid‐rifampicin composites have since been tested in a rabbit model, using a new bioluminescent imaging methodology for simpler preclinical evaluation of orthopaedic implant‐associated infections.^[184]^ Both the live bioluminescent imaging and the *ex vivo* colony forming unit data demonstrated that the drug loaded composite coatings were able to protect the animals from MRSA infection for up to 7 days. These exciting composite fibre materials offer significant advantages in tunability of multiple drug loading and release, compared to single formulation coating technologies discussed previously (section 7.3).

Regenerative tissue engineering brings together material science and stem‐cell engineering to provide optimised biological niches to enhance healing processes.^[185]^ One common example of these systems are mesenchymal stem cells (MSC), encapsulated within a hydrogel matrix that imitates the extracellular matrix of healthy tissues. While these materials can be highly effective, they can be prone to biofilm related infections, like any other implanted device. Beneficially the hydrogel is a natural reservoir for nutrients, signalling molecules and therapeutics, and so antibiotics can be incorporated to reduce the risk from biofilms. A recent example by Kao and co‐workers^[186]^ has investigated composite hydrogels of polyethylene glycol and gelatin, loaded with a combination therapy of minocycline, vancomycin and linezolid, for the encapsulation of MSCs. The study investigated the effect of each antibiotic on a range of pro‐healing functions of the MSCs. In most cases it was found linezolid showed no benefit to any of these parameters, when used alone, but when used synergistically with the other two antibiotics an enhancement was seen. While combination therapy with linezolid and vancomycin did not improve the healing ability of MSCs, compared to monotherapy alone, they did improve the antibacterial efficacy. Previous work by the group showed that MSCs alone, and in combination with minocycline can only inhibit *S. aureus* infection up to 16 hours.^[187]^ The triple combination therapy was able to inhibit both planktonic bacterial growth, as well as adhesion to the hydrogel surface up to final timepoint of 18 hours.^[186]^ While this preliminary result on adhesion is promising, a more thorough investigation of the anti‐biofilm properties of these materials, and the role of the three antibiotics is needed for further development.

## 8 Summary and perspective

Very few oxazolidinone compounds have been tested against biofilms, yet biofilms are significant contributors to bacterial pathogenicity and virulence. Evidently, biofilms need to be considered when evaluating the activity and effectiveness of antibiotics.

The collection of compounds reviewed in this work with documented anti‐biofilm properties highlights the variety of laboratory testing methods used to determine activity against biofilms and the parameters that can be tested. Issues arise when attempting to compare the results of these assays as there is no standardisation across these methods. How is it possible to decipher which drug would be a good candidate to be taken further for clinical trials? This calls for the need to have standardized biofilm testing techniques and models (particularly for *in vitro* models). Efforts to date have focused on standardized methods for *P. aeruginosa* biofilms[^188^, ^189^] but there is still work to be done for other bacterial strains. Understandably, standardisation would be more difficult for *in vivo* biofilm models as different infection settings would require different methods of testing.^[69]^ Work by Coenye and Nelis^[69]^ has also emphasised the need to standardise the recovery of biofilm‐grown cells from surfaces to avoid artifacts and the introduction of air bubbles that cause excessive detachment.

Another major challenge associated with biofilm testing is the clinical relevance of the assays used. To what extent can the formation of a biofilm grown in the wells of microtiter plate clinically represent the formation of a biofilm on a wound or a catheter which has undergone serval rounds of decontamination? Often biofilms are grown from between 6–48 hours before testing is undertaken. How close does the maturity of the biofilms in these models mimic what is seen clinically? A key influence in the function of a biofilm is its thickness.^[190]^ However, reports on the anti‐biofilm activity of many of the oxazolidinone compounds considered in this review do not include reference to the thickness of the biofilms used in their studies. This calls into question whether the thickness of biofilms used is clinically relevant. These are very complex issues which are thoroughly explored in reviews by Stoodley^[191]^ and Vyas.^[192]^

The identification and design of oxazolidinone compounds with improved anti‐biofilm activity does not only rely on clinically relevant models but also a better understanding of their mode of action. Currently, the exact mechanism for the inhibition and eradication of biofilms by oxazolidinone agents is unknown. The compounds reviewed above suggest that there is no logic behind why some oxazolidinone compounds have better anti‐biofilm activity than others. Hopefully more research into the mechanism of action of oxazolidinones against biofilms will accelerate the discovery of new anti‐biofilm oxazolidinone agents. Computational methods can help to advance this area of research. Hancock and co‐workers^[71]^ describe the use of proteomics and transcriptomics to find targets for anti‐biofilm agents. *In silico* screening can also help, however molecular docking experiments to identify oxazolidinone compounds with good activity in terms of MIC is difficult due to the flexible nature of RNA.^[193]^ This becomes even more difficult when anti‐biofilm activity is also taken into account without a clear target. Perhaps a better route would be the identification of potent compounds through machine learning, as this approach relies on the properties of the molecule rather than the target. It is important to note that machine learning and other *in silico* techniques would require large datasets of anti‐biofilm activity data, which also calls for a standardisation of efficacy in biofilm testing. Right now, there are not enough oxazolidinone compounds being tested in biofilms to ensure oxazolidinone agents selected from these approaches would be effective in humans.

In conclusion, to improve the potency of oxazolidinones towards bacterial biofilms, we need to consider biofilms when undertaking antimicrobial testing. Therefore, we need to have a standardised method and model of evaluating anti‐biofilm activity that is clinically relevant. This, in turn, will help with the integration of *in silico* methods in oxazolidinone drug discovery and improve the potency of oxazolidinone‐based compounds towards biofilms as a result.

## Conflict of interest

The authors declare that they have no known competing financial interests or personal relationships that could have appeared to influence the work reported in this paper.

## Biographical Information

*Audrey R. N. Ndukwe received her B.Sc (Biochemistry) degree from the University of Essex and her M.Sc in Drug Design and Discovery from the University of Salford. She is now a Ph.D. Candidate at the Queensland University of Technology's Centre for Materials Science under the supervision of Kathryn E. Fairfull‐Smith. Her research project focuses on the development of novel anti‐biofilm agents*.

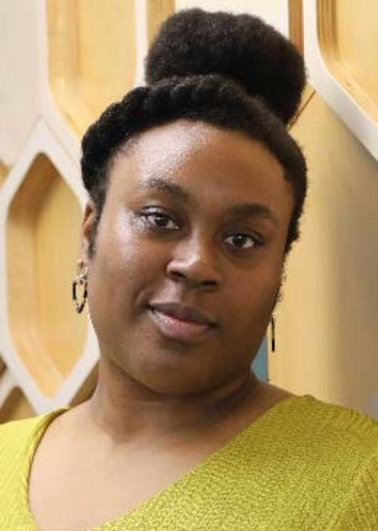

## Biographical Information

*Sandra Wiedbrauk received her Ph.D. degree in organic chemistry from Ludwig‐Maximilians‐Universität in Munich in 2018 under the supervision of Prof. Henry Dube. In 2018 she joined Queensland University of Technology as a postdoctoral fellow to work on advanced materials. Since 2021 she is working in the research group of Kathryn E. Fairfull‐Smith on anti‐biofilm molecules*.

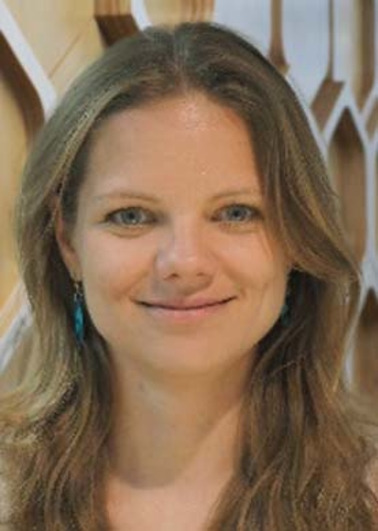

## Biographical Information

*Nathan R. B. Boase is a senior lecturer at the Queensland University of Technology and a member of the Centre for Materials Science. He completed his Ph.D. at the University of Queensland in 2015, under the supervision of Prof. Kristofer Thurecht. In 2019 he was recognized as a CAS Future Leader in chemistry. His current research explores controlled synthetic strategies to design materials that can respond to their environment. These materials are applied to solve significant challenges in healthcare, such as antibiotic coatings, nanomedicine, and antiviral therapies*.

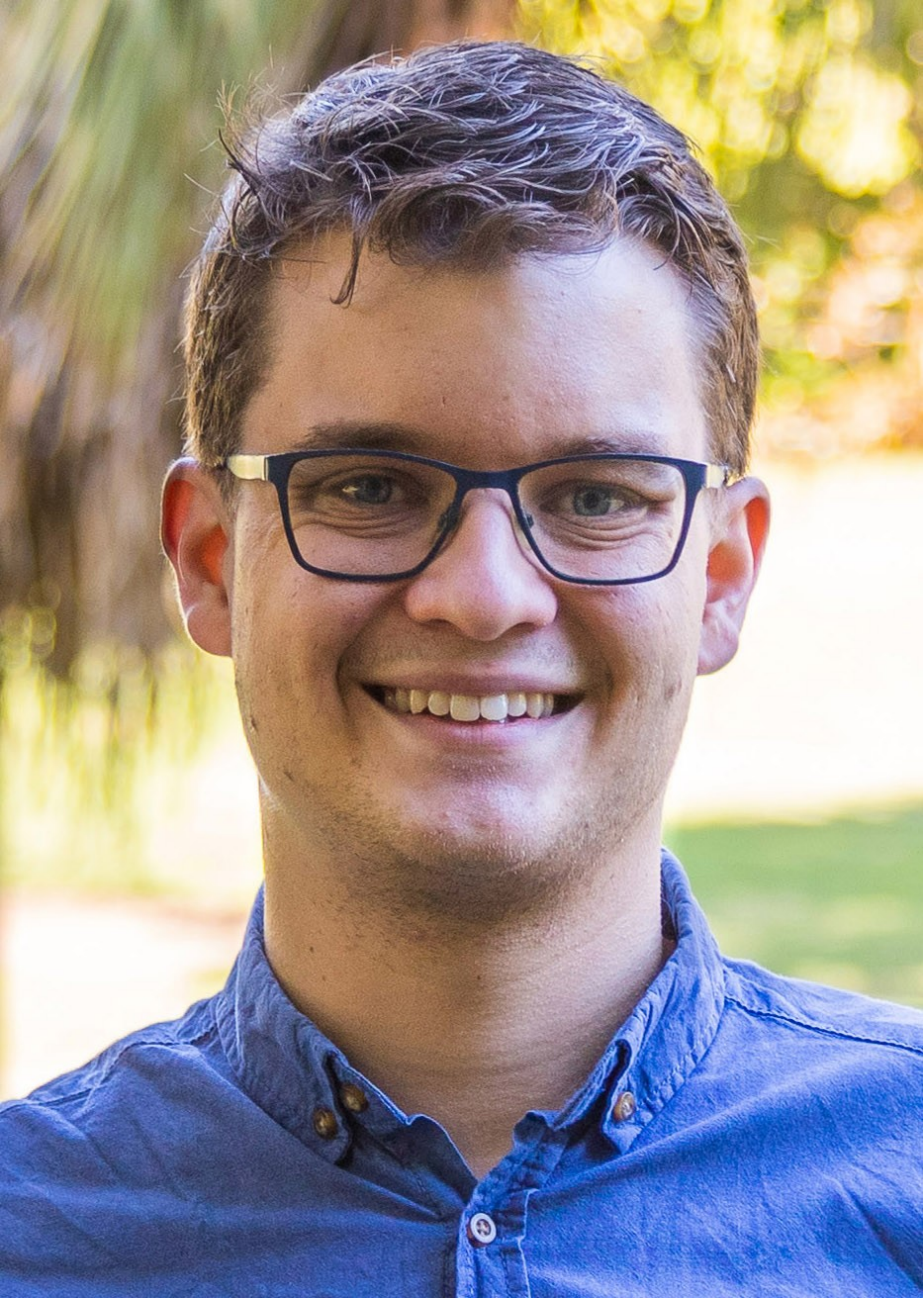

## Biographical Information

*Kathryn E. Fairfull‐Smith received her Ph.D. degree in organic chemistry from Griffith University in 2004. She worked as a post‐doctoral fellow at the University of Sheffield, England, and Queensland University of Technology (QUT) before beginning her independent academic career at QUT in 2009. She is currently an ARC Future Fellow and a Director of the QUT Centre for Materials Science. Her research focuses on the study of free radicals and reactive oxygen species in a range of applications, including the development of novel anti‐biofilm agents and materials*.